# Disease control in patients with non‐small cell lung cancer using pemetrexed: Investigating the best treatment strategy

**DOI:** 10.1111/1759-7714.15286

**Published:** 2024-03-14

**Authors:** Yutaka Takahara, Ryudai Abe, Nagae Sumito, Takuya Tanaka, Yoko Ishige, Ikuyo Shionoya, Kouichi Yamamura, Kazuaki Nishiki, Masafumi Nojiri, Ryo Kato, Shohei Shinomiya, Taku Oikawa

**Affiliations:** ^1^ Department of Respiratory Medicine Kanazawa Medical University Kahoku‐gun Japan

**Keywords:** bevacizumab, chemotherapy, non‐small cell lung cancer, pemetrexed, TTF‐1

## Abstract

**Background:**

Pemetrexed (PEM) is the primary chemotherapy for non‐small cell lung cancer (NSCLC), showing potential for long‐term disease stability in certain cases. However, studies examining disease control with PEM therapy are lacking. This study aimed to pinpoint clinical traits in patients with NSCLC responding well to PEM therapy, predict factors influencing disease control, and suggest optimal treatment approaches.

**Methods:**

A retrospective analysis of patients with NSCLC treated with PEM was performed to compare patients who achieved disease control after treatment with those who did not.

**Results:**

Of 73 patients, 56 (76.7%) achieved disease control with PEM therapy. In the disease control group, a significantly higher proportion of patients exhibited good performance status (PS) and received PEM doses without reduction after the second cycle. Multivariate analysis identified bevacizumab (Bev) noncompliance, PEM dose reduction, and thyroid transcription factor‐1 (TTF‐1) negativity as significant independent risk factors for disease progression during PEM therapy. Additionally, overall survival was significantly longer in the disease control group (*p* < 0.001).

**Conclusions:**

Our findings indicated that maintaining the dose of PEM after the second treatment cycle in patients with NSCLC, along with concurrent use of Bev and the presence of TTF‐1 positivity, could enhance disease control rates and extend survival.

## Graphical abstract

This study aimed to pinpoint clinical traits in patients with non‐small cell lung cancer (NSCLC) responding well to pemetrexed (PEM) therapy, predict factors influencing disease control, and suggest optimal treatment approaches. Our findings indicated that maintaining the dose of PEM after the second treatment cycle in patients with NSCLC, along with concurrent use of Bev and the presence of TTF‐1 positivity, could enhance disease control rates and extend survival.

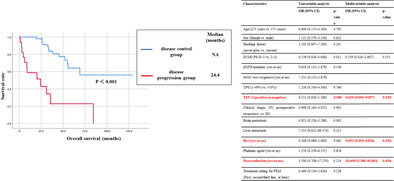

## INTRODUCTION

Pemetrexed (PEM)‐based chemotherapy is the recommended standard treatment regimen for primary, maintenance, and secondary therapy in patients with nonsquamous non‐small cell lung cancer (NSCLC).^1^, ^2^, ^3^ Maintenance therapy with PEM typically consists of a median of four cycles.^3^ However, some patients experience disease stability even after prolonged PEM administration.^1^

Previous studies have identified factors influencing PEM therapy outcomes, including sex,^4^ smoking,^5^, ^6^ epidermal growth factor receptor (EGFR) mutations,^5^, ^6^, ^7^, ^8^, ^9^, ^10^ anaplastic lymphoma kinase (ALK) gene rearrangements,^5^, ^6^, ^7^, ^8^, ^9^, ^10^ ROS1 gene rearrangements,^11^, ^12^, ^13^, ^14^, ^15^ and thyroid transcription factor‐1 (TTF‐1) expression.^16^, ^17^

However, these studies investigated the efficacy of PEM without considering the differences in the timing of treatment or concomitant therapy. We previously reported the potential contribution of maintaining treatment intensity to disease control and prolonged survival in maintenance therapy for lung cancer.^18^, ^19^ Therefore, we hypothesized that maintaining the treatment intensity might improve disease control in PEM therapy.

This study aimed to pinpoint clinical traits in patients with NSCLC responding well to PEM therapy, predict factors influencing disease control, and suggest optimal treatment approaches. This study compared patients with NSCLC treated with PEM therapy who achieved disease control with those who did not.

## METHODS

Patients with advanced‐stage (stage IIIB and IV, postoperative recurrence) NSCLC treated with PEM between August 2012 and December 2022 were retrospectively identified.

Data on age, sex, smoking history, performance status (PS), lung cancer histology, tumor proportion score (TPS), EGFR gene mutations, ALK rearrangements, ROS1 gene rearrangements, and clinical stage were collected during PEM therapy.

Responses were evaluated using the Response Evaluation Criteria in Solid Tumors (RECIST; version 1.1). Patients with partial response (PR) and stable disease (SD) were classified into the disease control group (*n* = 56), whereas patients with progressive disease (PD) were classified into the disease progression group (*n* = 17). Clinical information between the two groups was compared.

This study aimed to determine the effects of PEM therapy on disease control. Patients who received PEM plus immune checkpoint inhibitors were excluded from the study.

### Statistical analysis

All analyses were performed using the statistical software SPSS 26.0 (SPSS Inc.). Statistical significance was set at *p* < 0.05 (two‐tailed). All categorical variables were analyzed using the chi‐square test, whereas those with a predicted frequency below five were analyzed using Fisher's exact probability test. A *t*‐test without correspondence was used to compare the means of continuous variables between the two groups. A multivariate analysis was performed using logistic regression.

Survival curves were generated using the Kaplan–Meier method, and analyses were performed from the start of lung cancer treatment until death or the end of treatment. Survival analysis was performed up to mid‐December 2023.

The log‐rank test was employed to assess survival differences in response to PEM therapy. Statistical significance was considered when the risk rate was below 5%. This study was approved by the Review Committee of Kanazawa Medical University Hospital (approval no.: C057).

## RESULTS

### Study cohort

A total of 73 patients were identified. A total of 11 patients in the study received PEM monotherapy: four (36.4%) patients achieved PR, four (36.4%) had SD, and three (27.3%) experienced PD. The response and disease control rates were 36.4% and 72.7%, respectively. A total of 29 patients received carboplatin (CBDCA) + PEM therapy: 11 (37.9%) patients demonstrated a PR, eight (27.6%) had SD, and 10 (34.5%) showed PD. The response and disease control rates were 37.9% and 65.5%, respectively A total of 33 patients received platinum + PEM + bevacizumab (Bev) therapy: 13 (39.4%) patients achieved PR, 16 (48.5%) had SD, and four (12.1%) experienced PD. The response and disease control rates were 37.9% and 87.9%, respectively. No patients received PEM + Bev therapy as initial therapy. Disease control was achieved in 56 (76.7%) patients following PEM therapy (disease control group), whereas 17 (23.3%) were classified in the disease progression group.

The patient characteristics are presented in Table 1. Patients with good PS were significantly more prevalent in the disease (*p* = 0.005). After the second cycle, the dose of PEM was lowered in 10 patients, with nine of them undergoing a dose reduction during the second cycle and one during the third cycle. The reasons for the PEM dose reduction were thrombocytopenia (grade 4), anorexia (one grade 3 and four grade 2), WBC reduction (two grade 2), and neutropenia (one grade 4 and one grade 3). In contrast, more patients in the disease progression group received a reduced PEM dose after the second cycle (*p* < 0.001). The duration of PEM therapy was significantly longer in the disease control group (13.3 cycles vs. 2.1 cycles, *p* < 0.001).

**TABLE 1:** Comparison of patient characteristics between response and nonresponse groups.

	Disease control	Disease progression	*p*‐value
*n* (%)	56 (76.7%)	17 (23.3%)	
Age (years), mean (range)	65.7 (29–81)	62.7 (20–78)	0.414
Sex (male/female)	28/28	8/9	0.832
Smoking history (never/prior, current)	34/22	13/4	0.235
ECOG PS (0–1/2–4)	53/3	12/5	0.005*
Clinical stages (IV, postoperative recurrence/III)	50/6	15/2	1.000
Distant metastasis (yes/no)			
Brain metastasis	14/42	4/13	0.902
Liver metastasis	1/55	2/15	0.133
Pleural metastasis	24/32	5/12	0.321
*EGFR* mutation (yes/no)	25/31	10/7	0.305
*ALK* gene rearrangements (yes/no)	3/53	0/17	1.000
*ROS1* gene rearrangements (yes/no)	1/55	2/15	0.1330
PDL1 expression (22C3) (%), (0–49/50–100/ untested)	26/12/18	7/4/6	0.930
TTF‐1 (yes/no/untested)	32/2/22	9/3/5	0.125
Number of cycles of PEM therapy, mean (range)	13.3 (2–70)	2.1 (1–4)	<0.001^b^
Treatment setting for PEM (first‐, second‐/third‐line, or later)	9/47	5/12	0.221
ICIs in the immediate prior regimen (yes/no)	9/47	0/17	0.105
Dose reduction (yes/no/one cycle only)	7/49/0	3/6/8	<0.001^a^
Bev (yes/no)	28/28	4/13	0.054
Platinum drugs (yes/no)	48/8	15/2	1.000

Abbreviations: ALK, anaplastic lymphoma kinase; Bev, bevacizumab; ECOG, Eastern Cooperative Oncology Group performance status; EGFR; epidermal growth factor receptor; ICIs, immune checkpoint inhibitors; PDL1, programmed death ligand 1; PEM, pemetrexed; PS, performance status; TTF‐1, thyroid transcription factor‐1.

* *p* < 0.05.

^a^ Based on Fisher's exact test.

^b^ Based on the unpaired *t*‐test.

Patients who received PEM therapy in combination with a platinum agent did not differ significantly between the two groups. Similarly, patients who received PEM with Bev did not differ significantly between the two groups. Moreover, age, sex, smoking history, programmed death ligand 1 expression, TTF‐1, driver gene mutations, clinical stage, and treatment duration showed no significant differences between the two groups.

In this study, the rate of discontinuation of PEM therapy owing to adverse events was 17.8% (13/73). Table 2 presents the characteristics of patients who ceased PEM therapy due to adverse events. Of these adverse events, five were classified as grade 3, whereas the remaining eight were grade 2, all resolved solely by discontinuing PEM. Among these patients, three underwent PEM dose reduction due to side effects, specifically fatigue (grade 2) and decreased white blood cell (WBC) count (grade 2 in one patient and grade 4 in another). However, patients in Cases 2 and 5 continued PEM treatment with dose reduction. Finally, the patient in Case 2 had PEM treatment interrupted owing to the development of grade 2 conjunctivitis, whereas the patient in Case 5 faced interruption due to grade 3 renal dysfunction. Case 3 patient's PEM dose was reduced and continued owing to fatigue; however, the patient's symptoms did not improve, and treatment was discontinued by the attending physician after the 10th cycle of PEM therapy.

**TABLE 2:** Patients with PEM therapy discontinued due to adverse effects.

Case	Age/sex	AE (grade)	Treatment line	Previous treatment	Initial treatment (cycle)	Maintenance (cycle)	Dose reduction (reason)
1	68/M	Anemia (2)	Second	Pembrolizumab monotherapy	CBDCA + PEM (4)	PEM (11)	None
2	70/M	Conjunctivitis (2)	First	None	CBDCA + PEM + Bev (4)	None	WBC decreased
3	71/F	Malaise (2)	Second	Afatinib	CBDCA + PEM (2)	PEM (10)	Malaise
4	69/M	Pneumonitis (2)	First	None	CDDP + PEM + Bev (4)	PEM (2)	None
5	68/F	Renal dysfunction (3)	Second	Alectinib	CBDCA + PEM + Bev (4)	PEM + Bev (4)	WBC decreased
6	71/F	Anemia (3)	First	None	CDDP + PEM (4)	PEM (3)	None
7	29/F	Platelet count decreased (2)	First	None	CDDP + PEM + Bev (5)	PEM + Bev (2)	None
8	64/M	Infection (3)	Second	CBDCA + PTX + Bev + atezolizumab	CDDP + PEM + Bev (4)	None	None
9	75/M	Purpura (3)	First	None	CBDCA + PEM + Bev (6)	PEM + Bev (4)	None
10	69/M	Pneumonitis (3)	Second	CBDCA + PTX + Bev + atezolizumab	CDDP + PEM + Bev (4)	None	None
11	70/F	Malaise (2)	First	None	PEM (20)		None
12	77/F	Renal dysfunction (3)	First	None	CBDCA + PEM (6)	PEM (3)	None
13	70/M	Pneumothorax (2)	Second	CBDCA + PTX + Bev	PEM (5)	PEM + Bev (5)	None

Abbreviations: AE, adverse effects; Bev, bevacizumab; CBDCA, carboplatin; F, female; M, male; PEM, pemetrexed; PTX, paclitaxel; WBC, white blood cell.

Univariate and multivariate analyses were performed to identify the risk factors for disease progression in PEM therapy (Table 3). In the univariate model, statistically significant risk factors predicting disease progression were poor PS (*p* = 0.012, odds ratio [OR], 0.139, 95% confidence interval [CI]: 0.028–0.648). Multivariate analysis showed that the noncombination of Bev (*p* = 0.036; OR, 0.053; 95% CI: 0.003–0.820), PEM dose reduction after the second cycle (*p* = 0.034; OR, 10.499; 95% CI: 1.189–92.691), and TTF‐1 negativity (*p* < 0.028; OR, 0.029; 95% CI: 0.001–0.697) were statistically significant risk factors for disease progression.

**TABLE 3:** Univariate and multivariate analyses of risk factors for disease progression in patients with NSCLC treated with PEM therapy.

Characteristics	Univariable analysis		Multivariable analysis	
	OR (95% CI)	*p*‐value	OR (95% CI)	*p*‐value
Age (≥75 years vs. <75 years)	0.800 (0.153–4.184)	0.792		
Sex (female vs. male)	1.125 (0.379–3.336)	0.832		
Smoking history (never/prior vs. current)	2.103 (0.607–7.284)	0.241		
ECOG PS (0–1 vs. 2–3)	0.139 (0.028–0.648)	0.012	0.259 (0.026–2.607)	0.251
*EGFR* mutation (yes or no)	0.618 (0.133–2.879)	0.540		
*ROS1* rearrangement (yes or no)	7.333 (0.133–2.879)			
TPS (1%–49% vs. >50%)	1.238 (0.356–4.300)	0.766		
TTF‐1 (positive or negative)	0.152 (0.020–1.160)	0.069	0.029 (0.001–0.697)	0.028
Clinical stages (IV, postoperative recurrence vs. III)	0.900 (0.164–4.932)	0.903		
Brain metastasis	0.923 (0.258–3.298)	0.902		
Liver metastasis	7.333 (0.622–86.474)	0.113		
Bev (yes or no)	0.308 (0.089–1.060)	0.062	0.053 (0.003–0.820)	0.036
Platinum agent (yes or no)	1.250 (0.239–6.537)	0.856		
Dose reduction (yes or no)	3.500 (0.709–17.270)	0.124	10.499 (1.189–92.691)	0.034
Treatment setting for PEM (First, second/third line, or later)	0.460 (0.130–1.626)	0.228		

Abbreviations: ALK, anaplastic lymphoma kinase; Bev, bevacizumab; CI, confidence interval; ECOG, Eastern Cooperative Oncology Group performance status; EGFR; epidermal growth factor receptor; NSCLC, non‐small cell lung cancer; OR, odds ratio; PEM, pemetrexed; PS, performance status; TTF‐1, thyroid transcription factor‐1.

**FIGURE 1:**
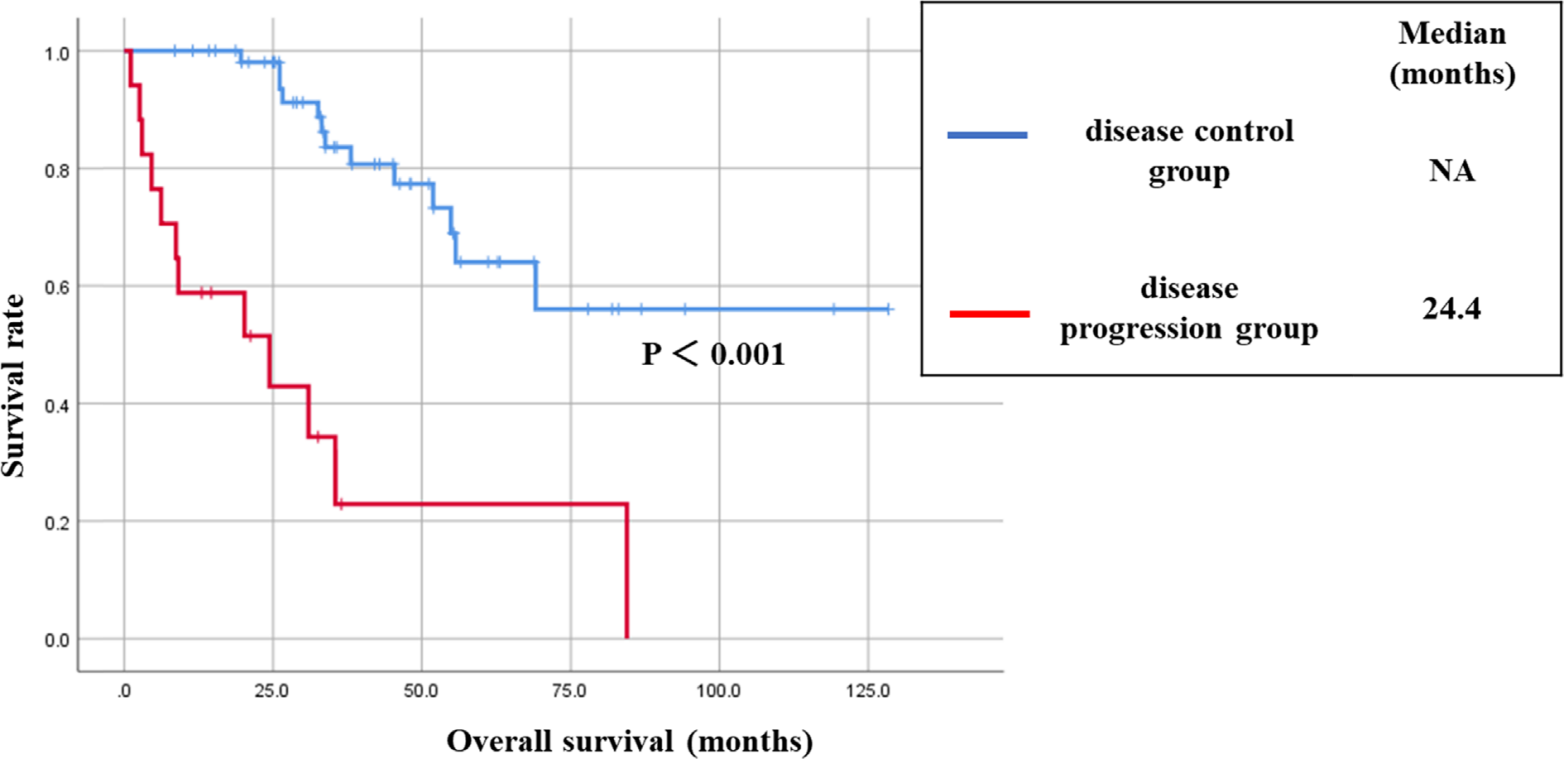
Overall survival in the disease control and the disease progression groups. The median survival in the disease control group was NA, whereas it was 24.4 months in the disease progression group, with a significant difference (log‐rank test, *p* < 0.001). NA, not applicable.

The survival curves for the disease control and disease progression groups are shown in Figure 1. A statistically significant survival benefit was observed in the response group (*p* < 0.001, log‐rank test).

## DISCUSSION

In this study, the PEM dose reduction after the second cycle was identified as a significant predictor of disease progression. Furthermore, the duration of PEM therapy was significantly longer in the disease control group, confirming that maintaining treatment intensity by avoiding PEM dose reduction after the second cycle of PEM therapy may contribute to disease control and prolonged survival.

In this study, 10 patients were identified who had reduced PEM therapy. Among them, one experienced a serious side effect (grade 4 thrombocytopenia), and the remaining nine had mild side effects, such as a decrease in WBC count and anorexia of grade 3 or less, suggesting that the combination of G‐CSF preparations^20^, ^21^ and supportive care^22^, ^23^, ^24^ could have allowed continuation of PEM therapy without dose reduction. However, the study as a whole found that 13 patients discontinued PEM therapy due to side effects (Table 2). Although no serious side effects of grade 4 or higher were observed, two patients developed pneumonia, and two experienced worsening renal function.

PEM can cause drug‐induced lung injury at a frequency of 0.8%–3.5%,^1^, ^25^ and deaths have been reported.^26^ Therefore, discontinuation of PEM should be considered at the onset of pneumonia, even in mild cases. Similarly, the risk of serious hematologic toxicity of PEM is increased in renal dysfunction.^27^, ^28^ Therefore, treatment with PEM is contraindicated in patients with a creatinine clearance of <45 mL/min.^29^ When such side effects occur, appropriate dose reduction or discontinuation of PEM may be necessary. However, the extent to which continued treatment without PEM dose reduction, with appropriate management such as concomitant supportive care, contributes to prolonged survival needs to be evaluated in future studies.

Since 2004, multiple studies have confirmed that Bev, in combination with platinum‐based treatment regimens, can prolong progression‐free survival (PFS) in patients with advanced nonsquamous NSCLC.^30^ Therefore, the National Comprehensive Cancer Network recommends platinum‐based chemotherapy combining Bev and PEM.^31^

Continued maintenance therapy after primary therapy for patients with nonsquamous NSCLC is now the standard of care. Three maintenance options after induction chemotherapy are available in clinical practice: Bev monotherapy, PEM monotherapy, or PEM + Bev therapy. However, an optimal maintenance strategy has not yet been determined.^32^

This study found that the combination of Bev predicted disease control in PEM therapy. The combination of Bev + PEM improves PFS and overall survival (OS) more than PEM monotherapy.^33^

Therefore, in patients for whom Bev is indicated, the combination of Bev should be considered in PEM maintenance therapy. However, the addition of Bev to PEM does not improve the prognosis in elderly patients.^34^ Additionally, a higher incidence of adverse effects has been reported with PEM + Bev maintenance therapy compared to PEM monotherapy.^35^, ^36^ Moreover, real‐world clinical practice includes patients for whom Bev is not applicable because of bleeding, renal dysfunction, arterial thrombosis, hypertensive crisis, poor wound healing, or gastrointestinal perforation.^37^ Thus, precise case selection is important for Bev combination therapy because of concerns regarding elderly patients and increased adverse effects.

In this study, TTF‐1 negativity was a predictor of disease progression following PEM therapy. Previous studies have reported a lower efficacy of chemotherapy with PEM in patients with TTF‐1‐negativity.^16^, ^38^, ^39^, ^40^, ^41^ This study indicated that TTF‐1 negativity was associated with disease progression in patients with lung cancer treated with PEM. Therefore, alternative treatment options should be considered for patients with TTF‐1‐negativity instead of PEM.

This study had several limitations. First, it was a single‐center retrospective study, potentially leading to bias in patient selection and data collection. The majority of patients were evaluated for response approximately 6–8 weeks after PEM therapy. However, due to its retrospective nature, these intervals were not as precisely defined as those in prospective studies. Notably, four patients in the disease progression group underwent an early CT after one cycle of PEM therapy to evaluate lesions 3 weeks post‐treatment due to suspected disease progression.

Additionally, few patients in this study tested positive for ALK and ROS1 gene rearrangements (three patients each). Particularly, the absence of patients with positive ALK gene rearrangements in the advanced disease group precluded their inclusion in the multivariate analysis.

Therefore, the findings of this study might be affected by insufficient statistical power to detect differences in ALK and ROS1 gene rearrangements. Future research should involve larger multicenter studies to mitigate this issue. Although the results of this study are preliminary, they do suggest an optimal treatment approach using PEM therapy for patients with NSCLC in real‐world clinical practice.

We believe that the results reported in this study will contribute to advancing clinicians' understanding of personalized treatment approaches in managing patients with NSCLC.

Second, the treatment protocols in this study, such as PEM reduction after the second cycle or concomitant Bev use, were not clearly defined and left to the discretion of attending physicians, potentially introducing bias among the study groups.

In this study, patients with a good PS were particularly numerous in the disease control group. Some patients in the disease control group may have responded to PEM therapy due to their good general condition, which cannot be dismissed. Future studies will require larger, prospective, multicenter studies to effectively address confounding factors and ascertain whether PEM therapy, particularly in combination with Bev and continued without dose reduction post the second cycle, genuinely exerts disease‐controlling effects.

In conclusion, the results suggest that in patients with TTF‐1‐negative NSCLC, continuing PEM therapy without dose reduction after the second cycle, combined with bevacizumab, improve disease control and prolong maintenance treatment and survival.

## AUTHOR CONTRIBUTIONS

All authors had full access to the study data and took responsibility for the integrity of the data and the accuracy of the data analysis. All authors read and approved the submission of this manuscript. Conceptualization: Yutaka Takahara. Resources: Yutaka Takahara, Ryudai Abe, Sumito Nagae, Takuya Tanaka, Yoko Ishige, Ikuyo Shionoya, Kouichi Yamamura, Kazuaki Nishiki, Masafumi Nojiri, Ryo Kato, Shohei Shinomiya and Taku Oikawa. Investigation: Ryudai Abe, Sumito Nagae, Takuya Tanaka, Yoko Ishige, Ikuyo Shionoya, Kouichi Yamamura, Kazuaki Nishiki, Masafumi Nojiri, Ryo Kato and Taku Oikawa. Methodology: Yutaka Takahara and Taku Oikawa. Writing–original draft preparation: Yutaka Takahara with support from Shohei Shinomiya and Taku Oikawa.

## FUNDING INFORMATION

This study received no specific grants from any funding agency in the public, commercial, or not‐for‐profit sector.

## CONFLICT OF INTEREST STATEMENT

The authors declare no conflicts of interest.

## Data Availability

The datasets used in this study are available from the corresponding author upon request.
